# Using the 4 M of the Age Friendly Health System to improve MIPS documentation in Primary care: A Feasibility study

**DOI:** 10.1093/geroni/igab046.3020

**Published:** 2021-12-17

**Authors:** Sweta Tewary, Denise Kruszynski, Naushira Pandya, Nicole Cook, Sashah Damier, Assma Twahir

**Affiliations:** 1 Nova Southeastern University, Fort Lauderdale, Florida, United States; 2 NSU, Davie,FL, Florida, United States; 3 NSU, Davie, Florida, United States

## Abstract

Age Friendly Health Systems (AFHS) commit to evidence-based, low-risk, coordinated care that is centered on what matters most to older adults, their families and caregivers. Nova Southeastern University South Florida Geriatric Workforce Enhancement Program (NSU SFGWEP) has partnered with multiple primary care clinics to provide dedicated AFHS training and support to increase AFHS transformation in Broward and Miami-Dade Counties. As part of the initiative, SFGWEP provide didactic training, clinic on-site brief demonstration, and infographic guidance for EHR documentation. NSU SFGWEP activities are conducted through training surveys, provider feedback, and e-clinical measures that align with CMS MIPS measures. Three participating health systems report annually on seven e-clinical measures that, collectively, provide indicators of the 4Ms of AFHS (what matters, medication management, mentation and mobility.) From baseline to Year 1, NSU SFGWEP saw improvement in controlled hypertension (54% to 94%), opioid screening (<1% to 11%), advance care planning (21% to 35%) and falls risk assessment (45% to 59%). Results demonstrate the need to continue and expand AFHS interventions for sustainability. In Year 2, SFGWEP will continue to expand awareness of best practices and benefits of the AFHS through education and training at NSU and at the various primary care sites. As mutual collaboration and implementation methods are shared among participating members, the expectation is that quality healthcare of our elder community adults will measurably improve.

